# *MBOAT7-TMC4* rs641738 Is Not Associated With the Risk of Hepatocellular Carcinoma or Persistent Hepatitis B Infection

**DOI:** 10.3389/fonc.2021.639438

**Published:** 2021-05-25

**Authors:** Peng Wang, Ying Li, Lu Li, Rong Zhong, Na Shen

**Affiliations:** ^1^ Institute and Department of Infectious Disease, Tongji Hospital, Tongji Medical College, Huazhong University of Science and Technology, Wuhan, China; ^2^ Department of Laboratory Medicine, Tongji Hospital, Tongji Medical College, Huazhong University of Science and Technology, Wuhan, China; ^3^ Research Center for Translational Medicine, Shantou University Medical College, Shantou, China; ^4^ Department of Epidemiology and Biostatistics, MOE Key Laboratory of Environment & Health, School of Public Health, Tongji Medical College, Huazhong University of Science and Technology, Wuhan, China

**Keywords:** hepatocellular carcinoma, persistent HBV infection, rs641738, susceptibility, MBOAT7 gene, case-control study

## Abstract

**Objective:**

A hot genetic variant, rs641738 within the *membrane-bound O-acyltransferase domain containing 7(MBOAT7)* and *transmembrane channel-like 4 (TMC4)*, was recently reported to be associated with several liver diseases. However, the results remain controversial. Therefore, this study aimed to explore the role of *MBOAT7-TMC4* rs641738 in the risk of hepatocellular carcinoma (HCC) and persistent hepatitis B virus (HBV) infection.

**Methods:**

We first conducted a case-control study that included 779 HCC cases and 1412 cancer-free controls. Controls consisted of 678 persistent HBV carriers and 734 spontaneously recovered subjects. The gene variant rs641738 was genotyped using the MassARRAY platform. The results were analyzed in five genetic models using multivariate logistic regression analyses. Next, we performed a systematic review and meta-analysis to further explore the role of this variant in HCC risk.

**Results:**

The results suggested no association between *MBOAT7-TMC4* rs641738 and HCC risk in most genetic models (all *P* > 0.05). Although a marginally significant association was observed in TT *vs*. CC (*P* = 0.037) and the recessive models (*P* = 0.044). The meta-analysis of 2135 HCC cases and 4388 controls supported that this variant was not related to HCC risk, even in the TT *vs*. CC and recessive models. We also determined that this variant did not influence persistent HBV infection.

**Conclusion:**

Our work highlights that *MBOAT7-TMC4* rs641738 is not associated with the risk of HCC or persistent HBV infection. This study provides some clues to identify the “truth” of potential disease-related genetic factors in the post-genome era.

## Introduction

Hepatocellular carcinoma (HCC) is a common rapidly progressing cancer, with high mortality worldwide, especially in China (1). As the fourth most common cancer in China, 466,100 new cases were estimated in 2015 (2). Environmental factors such as hepatitis B virus (HBV) infection have been confirmed to be crucial in the pathogenesis of HCC (3). Increasing evidence has revealed that genetic factors also play an important role in the development of HCC (4).

HCC is a complicated disease with high genetic heterogeneity. Candidate gene studies and genome-wide association studies (GWASs) have identified hundreds of genes and loci associated with HCC risk. How to identify the “truth” of these genetic factors is becoming an urgent issue in the post-genome era. In 2015, a GWAS study reported that a genetic variant near the *membrane-bound O-acyltransferase domain containing 7(MBOAT7)* gene, rs641738, C>T, increased the risk of alcohol-related cirrhosis (5). It is also reported to be located in the *transmembrane channel-like 4 (TMC4)* gene, which is not abundantly expressed in the human liver (6). The rs641738 variant is mapped to 500-bp downstream of *MBOAT7* and is located in exon 1 of the *TMC4* gene. Helsley et al. first reported that genetic deletion of Tmc4 in mice may not lead to hepatic steatosis, but the loss of function of its neighboring gene Mboat7 could result in liver disease progression in mice (7). Therefore, the rs641738 variant is often referred to as the *MBOAT7* or *MBOAT7-TMC4* variant. In 2016, Thabet et al. demonstrated that *MBOAT7-TMC4* rs641738 could be a risk factor for hepatic inflammation and liver fibrosis (8). Subsequent studies further investigated the association between this variant and HCC risk, but led to conflicting conclusions (9–11).

Here, we first conducted a case-control study including 779 HCC cases and 1,412 cancer-free controls, aiming to explore the effects of *MBOAT7-TMC4* rs641738 on HCC risk in a Chinese population. We then performed a systematic review and meta-analysis to further explore the role of this variant and validate our results.

## Materials and Methods

### Study Subjects

This case-control study included 779 HCC cases and 1,412 cancer-free controls. The cases were pathologically confirmed and enrolled between January 2014 and June 2016 at Tongji Hospital of Huazhong University of Science and Technology (HUST), central China. The controls are consisted of 678 persistent HBV carriers and 734 spontaneously recovered subjects, who were recruited from a health screening in the same hospital during the same period as the cases were included. Persistent HBV carriers were people who were positive for both of the hepatitis B surface antigen (HBsAg) and hepatitis B core antibody (HBcAb), but negative for antibody against hepatitis C virus (anti-HCV). Spontaneously recovered subjects were people who were negative for both of HBsAg and anti-HCV, but positive for both of HBcAb and hepatitis B surface antibody (HBsAb). All subjects were unrelated Han Chinese from Wuhan and the surrounding regions. Cases and controls were frequency-matched for sex and age (± 5 years). At recruitment, a 2-ml peripheral blood sample and a written informed consent were collected from each subject, and demographic information (i.e., sex, age, smoking and drinking status) were also obtained by questionnaire. The definitions of smoking and drinking status have been detailed previously (12, 13). Briefly, smokers were defined as subjects who smoked at least one cigarette a day and smoked for more than a year before the date of the interview; otherwise, they were defined as non-smokers. Drinkers were defined as subjects who drunk at least twice a week and drunk for more than a year before the date of the interview; otherwise, they were defined as non-drinkers. This study was approved by the institutional ethics committee of Tongji Hospital, Tongji Medical College of HUST.

### Serological Testing

Enzyme-linked immunosorbent assay (ELISA) was used to detect serum HBsAg, HBsAb, HBcAb, and anti-HCV (IMX; Abbott Diagnostics, USA). There were three positive controls, two negative controls, and one blank control in each reaction plate. About 5% of the samples were randomly chosen for repetition, and results were 100% concordant.

### Genotyping

Genomic DNA was extracted from leucocyte pellets of 2-ml peripheral blood by the coagulated blood DNA mini-extraction kit (DP6101, BioTeke Corporation, China). *MBOAT7-TMC4* rs641738 was genotyped using the Sequenom MassARRAY iPLEX Platform (SEQUENOM, CA, USA). All assays were conducted using a 384-well plate with positive and negative controls for each plate, without information on the disease status of the samples. We randomly selected 5% of the samples as duplicate sets and got a 100% concordance rate. The average call rate of this variant was 99.7%. A polar plot of rs641738 is shown in **Supplementary Figure S1**.

### Statistical Analysis

A goodness-of-fit χ^2^ test was applied to assess the Hardy-Weinberg equilibrium (HWE) in controls. Differences between cases and controls were examined by independent t-test, analysis of variance (ANOVA), or Pearson’s χ^2^ test according to the category of variables. The risk of HCC or persistent HBV infection was estimated by odds ratio (OR) and 95% confidence interval (CI), using a multivariate logistic regression analysis after adjusting for age, sex, smoking, and drinking status. Statistical significance was defined as a two-tailed *P* value < 0.05. All the analyses above were performed using IBM SPSS Statistics (version 20.0; Chicago, IL, USA). We calculated the statistical power of this study using Power V3.0 (14). Our sample size was determined based on an OR of 1.30 and a power of 0.95.

### Meta-analysis

This meta-analysis was performed according to the guidelines of the Preferred Reporting Items for Systematic Reviews and Meta-analyses (PRISMA) (15).

### Literature Search, Study Selection, and Data Extraction

A comprehensive literature search was conducted through PubMed, Embase, and Web of Science databases up to December 2020 without any restrictions. The search items included “*MBOAT7*,” “*TMC4*,” “rs641738,” and “hepatocellular carcinoma.” References from the identified publications were also reviewed to obtain potentially potential relevant studies.

The inclusion criteria were as follows: (1) a case-control or cohort study to evaluate the association between *MBOAT7-TMC4* rs641738 and HCC risk; (2) providing ORs and 95%CIs, or allele frequency and/or genotypes of this variant; and (3) the number of cases was more than 30. A study was excluded if it met one of the following criteria: (1) review, meta-analysis, comment, or conference abstract; (2) insufficient data to estimate OR and 95%CI; (3) a deviation from HWE in controls; and (4) studies with overlapping data. If a study contained overlapping data with another, we retained the study with a larger sample size.

Data were extracted from each included study as follows: first author, publication year, country, ethnicity, sex, age, sample size, genotype distribution, genotyping method, adjustment, and HWE information. Two authors independently assessed the studies as described above. Disagreements were resolved through discussion. The quality of each included study was evaluated through the Newcastle-Ottawa scale (NOS) (16). For case-control studies, the NOS has eight items, which are categorized into three dimensions: selection, comparability, and exposure. The score of NOS ranges from zero to nine and a study with score ≥ 7 is often considered as high-quality. Quality evaluation was not an exclusion criterion for eligible studies (**Supplementary Table S1**).

### Statistical Analysis for Meta-analysis

We used ORs and 95%CIs to estimate the association between *MBOAT7-TMC4* rs641738 and HCC risk. Multivariate-adjusted ORs and 95%CIs were preferentially extracted if available. Otherwise, the unadjusted ORs and 95%CIs were calculated instead. Between-study heterogeneity was examined using the Q test and *I^2^* statistic. If *P* < 0.10 or *I^2^* > 50%, significant heterogeneity was considered, and a random-effects model was applied. Otherwise, the fixed-effects model was used. To evaluate the quality, we performed a sensitivity analysis and publication test (17, 18). Sensitivity analysis was carried out by re-estimating pooled ORs and 95%CIs after excluding each eligible study in turn, aiming to assess the stability of the pooled results. The meta-analysis was conducted using Stata 12.0 software (College Station, TX, USA).

## Results

### Subject Characteristics

A total of 779 HCC cases, 678 persistent HBV carriers and 734 spontaneously recovered subjects were included in this study. Demographic characteristics are summarized in **Table 1**. In these three groups, the proportion of males in the three groups were 70.6%, 68.7%, and 68.1%, respectively. The mean ages were 53.20 ± 12.49, 52.27 ± 11.45, and 52.28 ± 12.89 years, respectively. There was no significant difference among the three groups on sex (*P* = 0.551), age (*P* = 0.240), smoking (*P* = 0.071), and drinking status (*P* = 0.091). Genotypes of *MBOAT7-TMC4* rs641738 in all groups did not deviate from HWE (HCC: *P* = 0.485; persistent HBV carriers: *P* = 0.133; spontaneously recovered subjects: *P* = 0.769).

**Table 1:** The characteristics of the included subjects.

Variables	HCC, N (%)	Persistent HBV carriers, N (%)	Spontaneously recovered subjects, N (%)	χ^2^/F	*P*
Total	779	678	734		
Sex				1.191	0.551
Male	550 (70.6)	466 (68.7)	500 (68.1)		
Female	229 (29.4)	212 (31.3)	234 (31.9)		
Age (mean ± SD)	53.20 ± 12.49	52.27 ± 11.45	52.28 ± 12.89	1.428	0.240
Smoking status				5.280	0.071
Smokers	301 (38.6)	243 (35.8)	242 (33.0)		
Non-smokers	478 (61.4)	435 (64.2)	492 (67.0)		
Drinking status				4.803	0.091
Drinkers	270 (34.7)	265 (39.1)	291 (39.6)		
Non-drinkers	509 (65.3)	413 (60.9)	443 (60.4)		

SD, standard deviation.

### Association of *MBOAT7-TMC4* rs641738 with Risk of HCC and Persistent HBV Infection

We performed four comparisons under the five genetic models, and the results are shown in **Table 2**. In the comparison of HCC cases versus (*vs*.) all controls, we found that after adjusting for sex, age, smoking, and drinking status, *MBOAT7-TMC4* rs641738 did not confer any increased risk of HCC in the dominant, additive, or allelic models (all *P* > 0.05). Although this variant seemed to be a risk factor for HCC in the two models, the results were marginally significant (TT *vs*. CC: *P* = 0.037; recessive: *P* = 0.044). Similar results were also observed in a subsequent comparison of HCC *vs*. persistent HBV carriers. When we set spontaneously recovered subjects as controls, we observed that *MBOAT7-TMC4* rs641738 was not associated with HCC risk (all *P* > 0.10). To explore whether this variant influenced the risk of persistent HBV infection, we conducted a further comparison between persistent HBV carriers and spontaneously recovered subjects. The results suggested that this variant was also unrelated to persistent infection or the clearance of HBV (all *P* > 0.30).

**Table 2:** The effects of *MBOAT7-TMC4* rs641738 on persistent HBV infection and HCC.

Genotypes	HCC, N (%)	Persistent HBV carriers, N (%)	Spontaneously recovered subjects, N (%)	Adjusted OR (95% CI), *P^a^*	Adjusted OR (95% CI), *P^b^*	Adjusted OR (95% CI), *P^c^*	Adjusted OR (95% CI), *P^d^*
CC	426 (54.7)	380 (56.1)	420 (57.7)	Reference	Reference	Reference	Reference
CT	295 (37.9)	264 (39.0)	264 (36.3)	1.05 (0.87–1.27), 0.604	1.00 (0.82–1.24), 0.981	1.10 (0.89–1.37), 0.370	1.11 (0.89–1.38), 0.366
TT	58 (7.4)	33 (4.9)	44 (6.0)	1.47 (1.02–2.12), 0.037	1.64 (1.04–2.57), 0.033	1.35 (0.89–2.05), 0.164	0.85 (0.53–1.36), 0.491
Dominant model				1.10 (0.92–1.32), 0.280	1.07 (0.87–1.32), 0.541	1.14 (0.93–1.40), 0.219	1.07 (0.87–1.32), 0.529
Recessive model				1.44 (1.01–2.06), 0.044	1.64 (1.05–2.55), 0.029	1.29 (0.86–1.95), 0.218	0.81 (0.51–1.29), 0.383
Additive model				1.13 (0.98–1.31), 0.094	1.13 (0.95–1.33), 0.172	1.13 (0.96–1.34), 0.138	1.02 (0.85–1.21), 0.851
Allelic model (T *vs*. C)				1.13 (0.98–1.30), 0.095	1.12 (0.95–1.33), 0.175	1.14 (0.96–1.34), 0.135	1.02 (0.86–1.21), 0.853

a HCC vs. (persistent HBV carriers + spontaneously recovered subjects), adjusted for sex, age, smoking, and drinking status.

b HCC vs. persistent HBV carriers, adjusted for sex, age, smoking,and drinking status.

c HCC vs. spontaneously recovered subjects, adjusted for sex, age, smoking, and drinking status.

d Persistent HBV carriers vs. spontaneously recovered subjects, adjusted for sex, age, smoking, and drinking status.

### Meta-Analysis of the Association Between *MBOAT7-TMC4* rs641738 and HCC Risk

**Figure 1:**
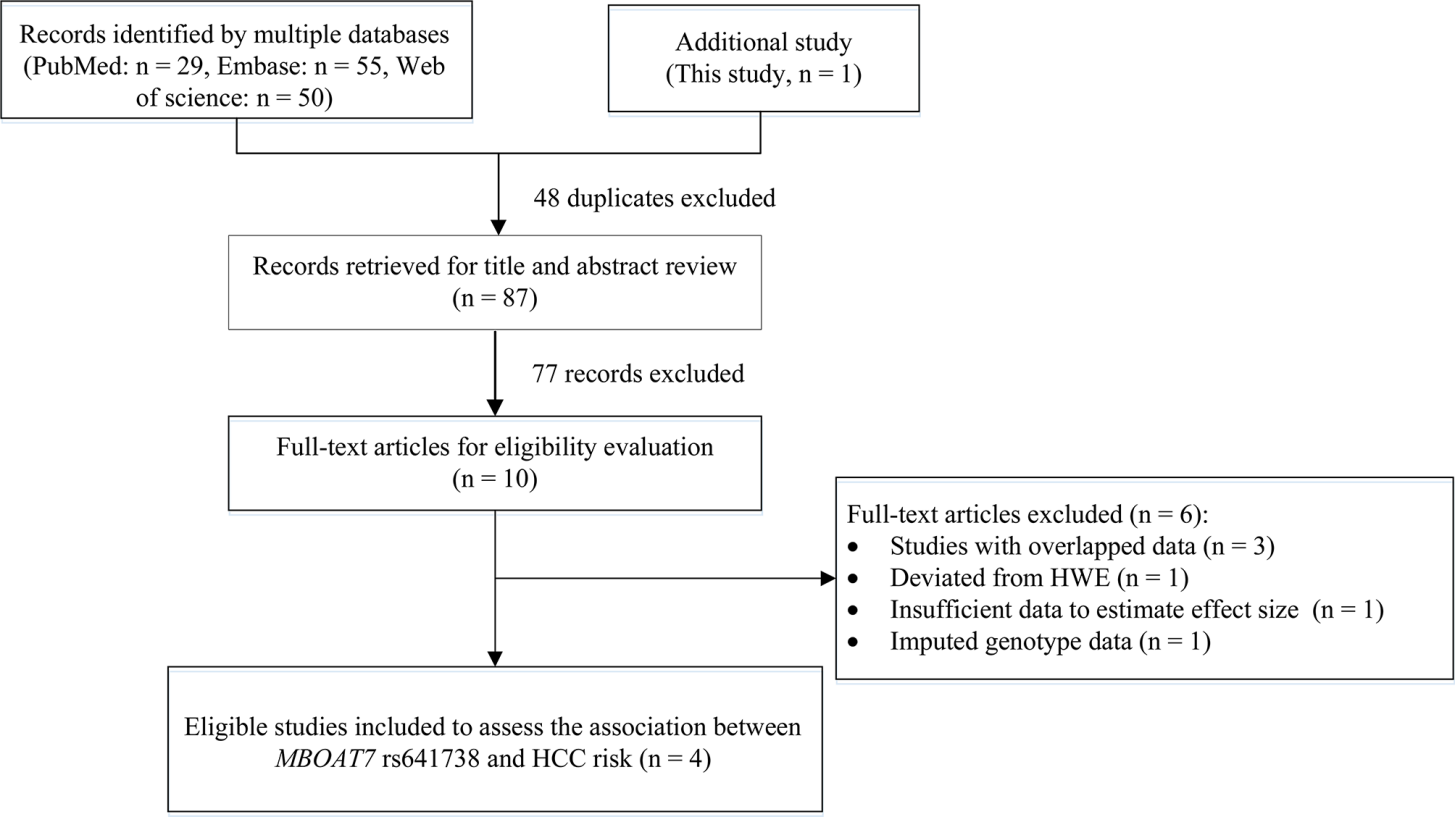
Flowchart of literature search and study selection.

We also conducted a systematic review and meta-analysis to further examine the results. Initially, we obtained 134 records by searching PubMed, Embase, and Web of Science databases, and excluded 48 duplicates and 77 records by title and abstract review. After reviewing the title and abstract and assessing the full-text articles, we excluded six studies. The study by Donati (2017) included two cohorts, the Italian non-alcoholic fatty liver disease (NAFLD) cohort and the UK NAFLD cohort (11). In the Italian NAFLD cohort: the rs641738 variant deviated from the HWE in controls (*P* = 0.031). In the UK NAFLD cohort, the number of HCC cases was too small (n = 20) to meet our inclusion criteria (n > 30). Therefore, we excluded this study from our meta-analysis. In addition, the study of Kawaguchi (2018) provided data on NAFLD type 4 and NASH-HCC rather than HCC data, therefore, we could not extract sufficient data to estimate the association between rs641738 and HCC risk and excluded this study (19). The remaining four studies were excluded because they had imputed genotype data (n = 1) (20) or overlapped data (n = 3) (21–23). Finally, we included three eligible articles (8–10) and this study for the meta-analysis (**Figure 1**).

**Figure 2:**
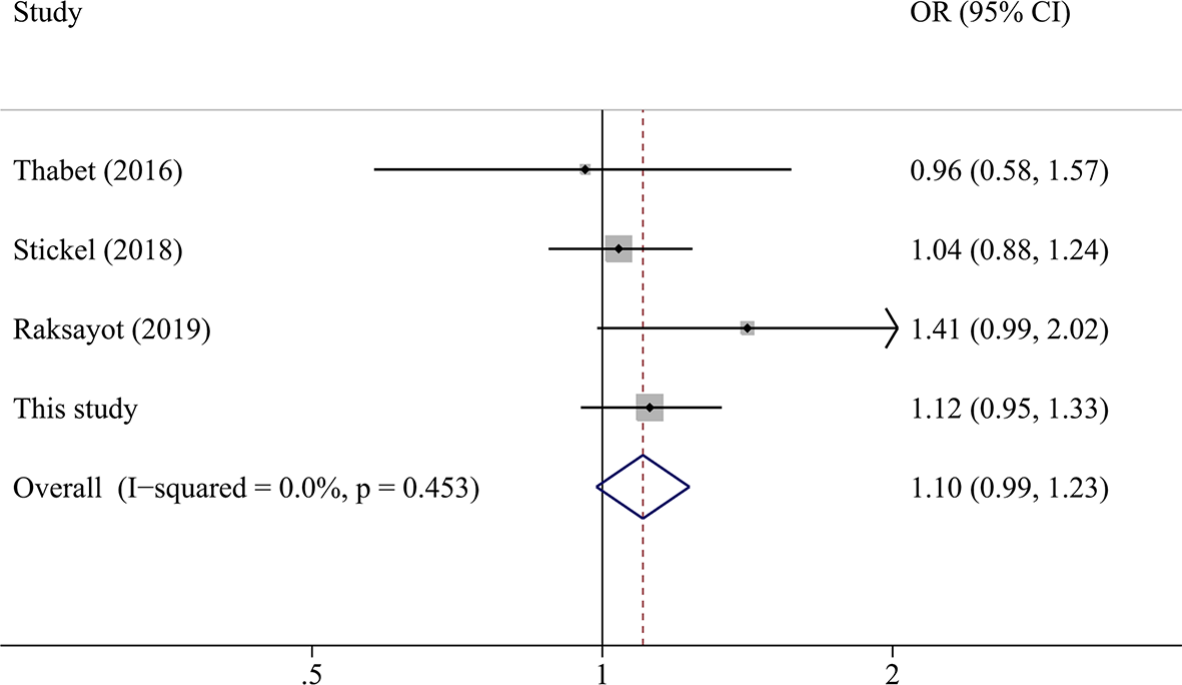
The forest plot of the association between *MBOAT7-TMC4* rs641738 and HCC risk under the allelic model.

A total of 2,135 HCC cases and 4,388 controls were included in the meta-analysis. Characteristics are detailed in **Table 3**. Except for the study by Raksayot (2019) (NOS score = 6) (10), all the studies were of high quality (NOS score ≥ 7). First, we performed a meta-analysis using the allelic model (**Figure 2**). The pooled results demonstrated that *MBOAT7-TMC4* rs641738 was not associated with HCC risk (OR = 1.10, 95% CI = 0.99–1.23, *P*_hetergeneity_ = 0.453, *I^2^* = 0%), which was stable suggested by the sensitivity analysis (**Supplementary Figure S2**). Egger’s or Begg’s test did not suggest any publication bias (*P*_Egger’s test_ = 0.723, *P*_Begg’s test_ = 1.000). Considering that marginally significant results were observed in our study, we performed meta-analyses using other common genetic models. As shown in **Table 4**, the pooled results in all these models also consistently showed that there was no association between *MBOAT7-TMC4* rs641738 and HCC risk. All pooled results were stable (**Supplementary Figure S2**) and showed no publication bias (**Table 4**).

**Table 3:** The characteristics of the included studies.

Study	Country	Ethnicity	Male, N (%)	Age (mean ± SD or mean [25th, 75th percentile])	Cases	Controls	Genotype (CC/TC/TT)		Genotyping method	Adjustment	HWE	NOS score*
							Case	Control				
Thabet (2016) (8)	Multi-country	Caucasian	Case: NA; Control: 1101 (64.5)	Case: NA; Control: 44.9 (38–52)	75	1706	24/35/16	16/531/822	TaqMan	Age, gender, BMI and Child-Pugh score	0.288	9
Stickel (2018) (9)	Switzerland	Mixed	Case: 679 (90); Control: 817 (70)	Case: 61 ± 10; Control: 55 ± 10	751	1165	203/363/185	185/314/583	TaqMan	Age, gender, BMI, and type II diabetes mellitus	0.934	7
Raksayot (2019) (10)	Thailand	NA	Case: 424 (0.8); Control: 77 (73.3)	HBV-HCC: 62.1 ± 7.8, HCV-HCC: 62.2 ± 7.5, NBNC-HCC: 63.3 ± 9.3; Control: 50.7 ± 4.6	530	105	279/213/38	38/66/34	TaqMan	No	0.818	6
This study	China	Chinese	Case: 550 (70.6); Control: 966 (68.4)	Case: 53.2 ± 12.5; Control: 52.3 ± 12.2	779	1412	426/295/58	58/800/528	Massary	Age, sex, smoking and drinking status	0.403	7

*NOS score ≥ 7 is often considered as high-quality.

**Table 4:** Meta-analysis of the association between *MBOAT7-TMC4* rs641738 and HCC risk under other genetic models.

Genetic model	OR (95%CI)	*P_hetergeneity_*	*I^2^* (%)	Sensitivity analysis	*P_Egger’s test_*	*P_Begg’s test_*
Codominant model (CT *vs*. CC)	1.04 (0.92–1.19)	0.394	0	Stable	0.591	1.000
Codominant model (TT *vs*. CC)	1.18 (0.97–1.43)	0.472	0	Stable	0.548	0.734
Dominant model	1.08 (0.95–1.22)	0.352	8.2	Stable	0.634	1.000
Recessive model	1.17 (0.98–1.38)	0.623	0	Stable	0.529	0.734
Additive model	1.08 (0.99–1.18)	0.367	5.1	Stable^*^	0.522	0.308

^*^Under the additive model, all pooled results were quite stable, except for excluding the study by Stickel (2018) (9). The pooled OR and 95% CI was 1.13 (1.00–1.28) when we excluded the study of Stickel (2018) (9) under the additive model.

## Discussion

We conducted a case-control study including 799 HCC cases and 1412 controls to investigate the effect of *MBOAT7-TMC4* rs641738 on HCC risk. Although this variant showed a marginally significant association in TT *vs*. CC (*P* = 0.037) and recessive model (*P* = 0.044), all other genetic models demonstrated non-significant results. We also performed a systematic review and meta-analysis to explore the role of *MBOAT7-TMC4* rs641738 in HCC susceptibility. A total of 2135 HCC cases and 4388 controls were included in the meta-analysis. The results consistently suggested that *MBOAT7-TMC4* rs641738 was not associated with HCC risk, even in both the TT *vs*. CC and recessive models. In addition, our study revealed that this variant was also not related to persistent HBV infection in any of the genetic models. To the best of our knowledge, it is the first study to investigate the association between *MBOAT7-TMC4* rs641738 and the risk of HCC and persistent HBV infection in Asians.

The rs641738 variant was first identified as a risk locus for alcohol-related cirrhosis (5) and was reported to be associated with nonalcoholic fatty liver disease (NAFLD)/nonalcoholic steatohepatitis (NASH) (24) and liver fibrosis (8). In 2016, Thabet et al. first investigated the association of *MBOAT7-TMC4* rs641738 with HCC risk, but did not find a significant result (8). Subsequently, Donati contradicted the conclusion and showed that this variant was predisposed to HCC in NAFLD subjects (11). However, a significant association was not confirmed in later studies (8–10). Even in the NAFLD cohort, *MBOAT7-TMC4* rs641738 did not show any association with the risk of Matteoni type 4 or NASH-HCC (19). Given the small sample size of the previous studies, we performed this case-control and meta-analysis and confirmed that *MBOAT7-TMC4* rs641738 was not associated with HCC risk.

Persistent HBV infection is a crucial risk factor for HCC in China. However, studies on the effect of *MBOAT7-TMC4* rs641738 on HBV infection are limited. Here, we also explored the association between *MBOAT7-TMC4* rs641738 and persistent HBV infection. The results exhibited that this variant was not related to spontaneous clearance of HBV, which concurred with a previous report from a Moroccan cohort (25). Interestingly, Thabet et al. reported that *MBOAT7-TMC4* rs641738 influenced hepatic inflammation and fibrosis in patients with persistent HBV infection (26). A possible explanation may be that the effect of this variant on liver disease is not associated with HBV or not a causal variant.

The role of *MBOAT7-TMC4* rs641738 in liver diseases is still conflicting. *MBOAT7* encodes a lysophosphatidylinositol acyltransferase involved in phospholipid metabolism. Some studies have shown that rs641738 reduces mRNA and hepatic MBOAT7 expression, whereas other studies have refuted this (11, 26, 27). Similar contradictions relating to fibrosis and NAFLD have also been reported (25–27). Whether and how *MBOAT7-TMC4* rs641738 influences liver diseases are still questionable. Despite this, our case-control study and meta-analysis suggest that this variant is not associated with the risk of HCC.

There are some limitations in our study. First, this was a hospital-based case-control study, which may have been influenced by selection bias. However, our multivariate analysis, power calculation, and subsequent meta-analysis ensured the accuracy of our conclusions. Second, our meta-analysis only included four studies because of the limited number of relevant studies. As such, we did not perform further stratified analyses according to factors including age, sex, smoking, or other lifestyle habits. Even so, the results of the meta-analysis are reliable because of robustness, no publication bias, and low heterogeneity. At last, some factors such as HBV viral load are also associated with HCC development, but this study did not collect the information and we failed to make further evaluation based on these factors. Future studies are needed to verify our results.

In summary, our case-control study and meta-analysis highlight that *MBOAT7-TMC4* rs641738 is not associated with HCC risk. Our work also suggests no relationship of this variant with persistent HBV infection in a Chinese population. Further studies are required to validate our results.

## Data Availability Statement

The original contributions presented in the study are included in the article and **Supplementary Material**, further inquiries can be directed to the corresponding author.

## Ethics Statement

The studies involving human participants were reviewed and approved by the institutional ethics committee of Tongji Hospital, Tongji Medical College of HUST. The patients/participants provided their written informed consent to participate in this study. Written informed consent was obtained from the individual(s) for the publication of any potentially identifiable images or data included in this article.

## Author Contributions

Conceptualization: NS. Methodology and data curation: PW and YL. Formal analysis: LL and RZ. Writing—original draft preparation: PW. Writing—review and editing: NS and RZ. All authors contributed to the article and approved the submitted version.

## Funding

This work was supported by National Natural Science Foundation of China (NSFC-81601839 to NS and NSFC-81602407 to LL).

## Conflict of Interest

The authors declare that the research was conducted in the absence of any commercial or financial relationships that could be construed as a potential conflict of interest.

## Abbreviations

*MBOAT7*, membrane bound O-acyltransferase domain containing 7; *TMC4*, transmembrane channel-like 4; HCC, hepatocellular carcinoma; HBV, hepatitis B virus; GWASs, genome-wide association studies; HUST, Huazhong University of Science and Technology; HBsAg, hepatitis B surface antigen; HBcAb, hepatitis B core antibody; anti-HCV, antibody against hepatitis C virus; HBsAb, hepatitis B surface antibody; ELISA, Enzyme-linked-immunosorbent assay; HWE, Hardy-Weinberg equilibrium; ANOVA, analysis of variance; OR, odds ratio; CI, confidence interval; PRISMA, Preferred Reporting Items for Systematic Review and Meta-analyses; NOS, Newcastle-Ottawa scale; vs., versus; NAFLD, nonalcoholic fatty liver disease; NASH, nonalcoholic steatohepatitis; SD, standard deviation.
