# Supplementary material for: MBOAT7-TMC4 rs641738 Is Not Associated With the Risk of Hepatocellular Carcinoma or Persistent Hepatitis B Infection
Source: Front Oncol. 2021 May 25;11:639438. doi: 10.3389/fonc.2021.639438 (PMC8185222; doi:10.3389/fonc.2021.639438)
Supplement: Supplementary file 1 [file DataSheet_1.pdf]

Supplementary Table S1. Quality evaluation of included studies by Newcastle-Ottawa Scale

Study	Selection (0-4)				Comparability (0-2)		Exposure (0-3)			Score
	CDA	RC	SC	DC	SCEA	AF	AE	SM	NR	
Thabet (2016)	1	1	1	1	1	1	1	1	1	9
Stickel (2018)	1	0	0	1	1	1	1	1	1	7
Raksayot (2019)	1	1	0	1	0	0	1	1	1	6
This study	1	0	0	1	1	1	1	1	1	7

Abbreviations: CDA, case definition adequate; RC, representativeness of the cases; SC, selection of controls; DC, definition of controls; SCEA, study controls for sex and age; AF, study controls for any additional factor; AE, ascertainment of exposure; SM, same method of ascertainment for cases and controls; NR, non-response rate.
