# Supplementary figures and images for: MBOAT7-TMC4 rs641738 Is Not Associated With the Risk of Hepatocellular Carcinoma or Persistent Hepatitis B Infection
Source: Front Oncol. 2021 May 25;11:639438. doi: 10.3389/fonc.2021.639438 (PMC8185222; doi:10.3389/fonc.2021.639438)

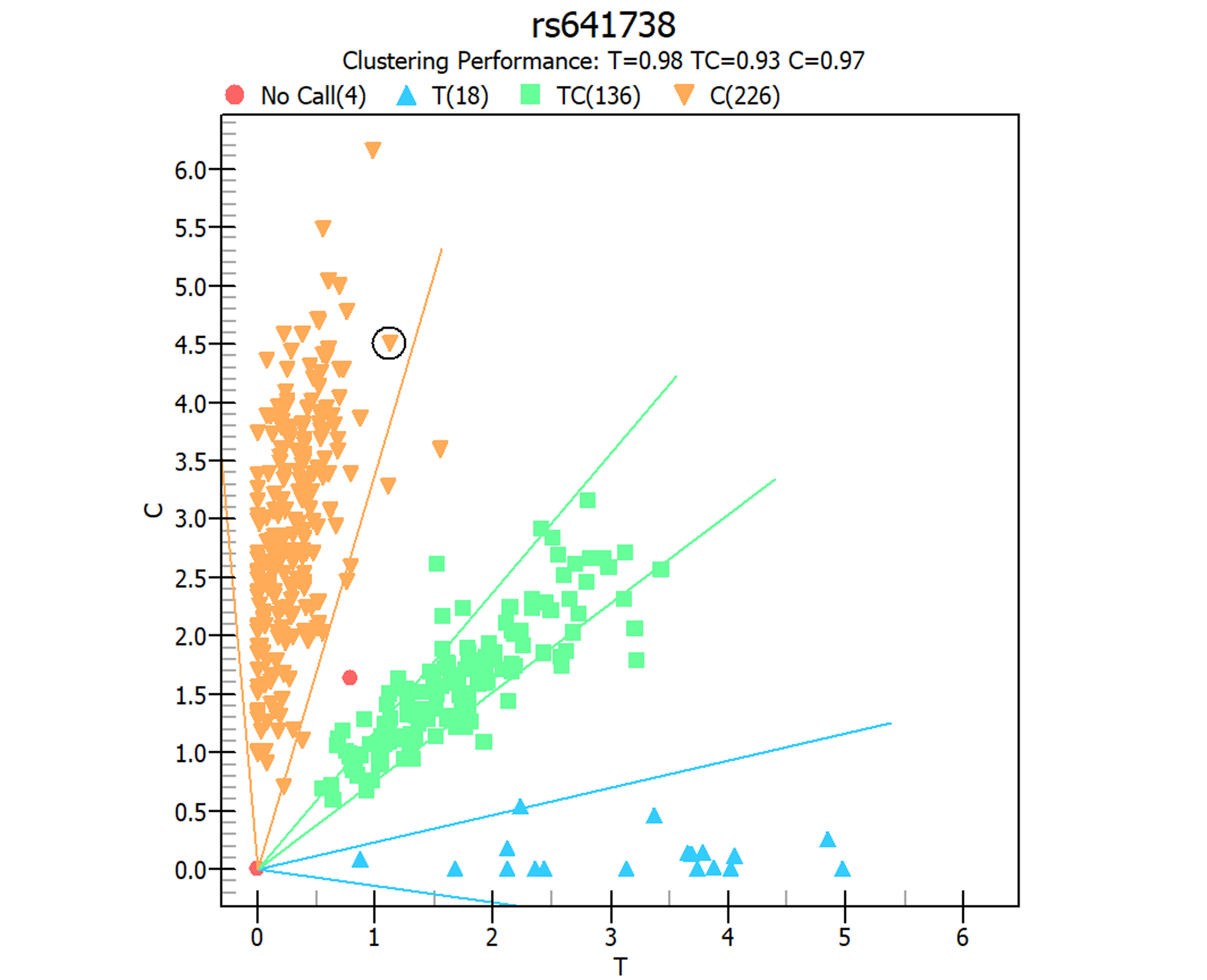

Supplement: Supplementary file 2 [file Image_1.tif]

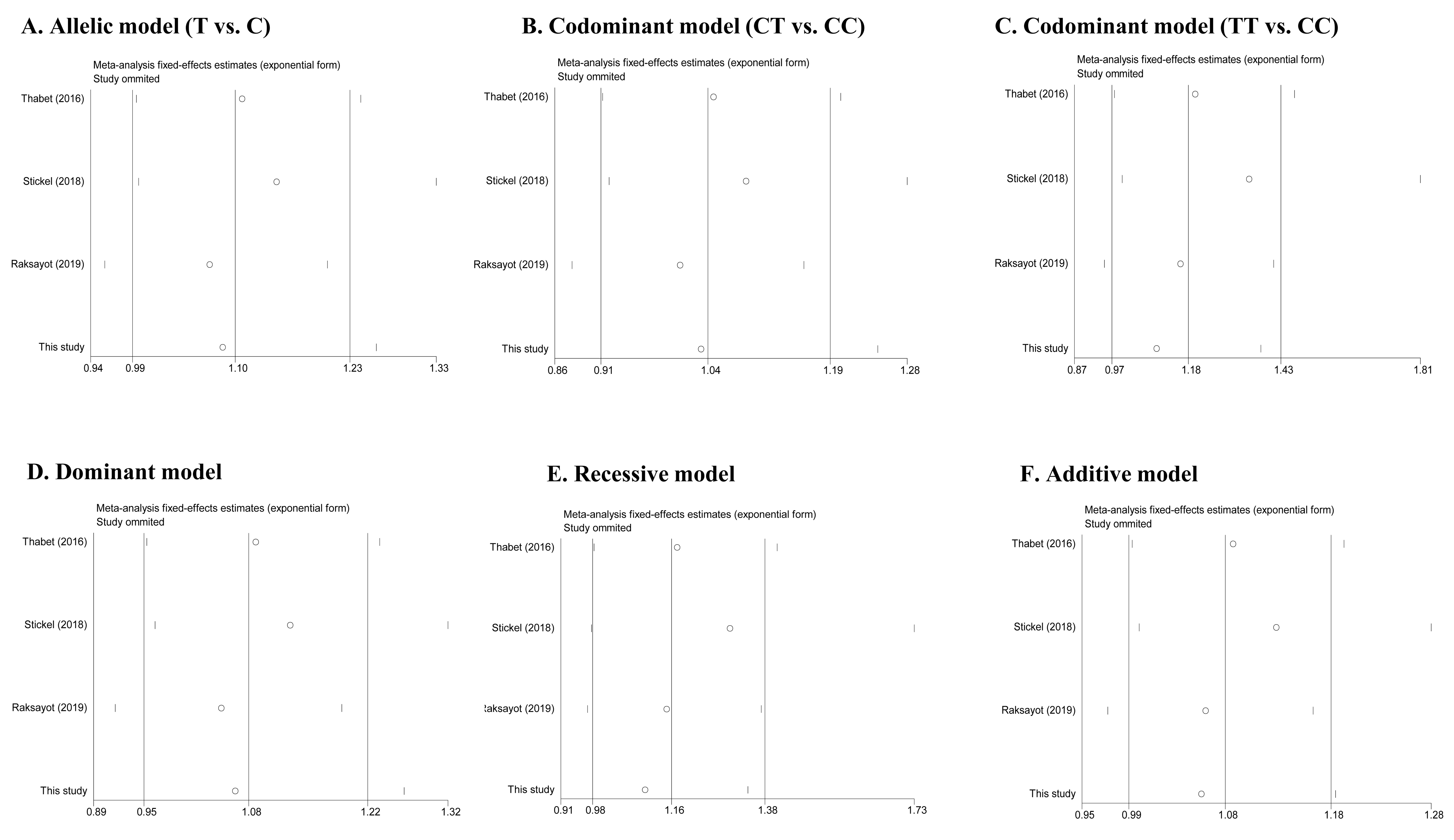

Supplement: Supplementary file 3 [file Image_2.tif]
